# Selection of Essential Medicines for Diabetes in Low and Middle Income Countries: A Survey of 32 National Essential Medicines Lists

**DOI:** 10.1371/journal.pone.0106072

**Published:** 2014-09-26

**Authors:** Yaser T. Bazargani, Anthonius de Boer, Hubert G. M. Leufkens, Aukje K. Mantel-Teeuwisse

**Affiliations:** Division of Pharmacoepidemiology and Clinical Pharmacology, Utrecht Institute for Pharmaceutical Sciences, Utrecht University, Utrecht, the Netherlands; University of Milan, Italy

## Abstract

**Aim:**

Diabetes is a growing burden especially in low and middle income countries (LMICs). Inadequate access to diabetes care is of particular concern and selection of appropriate diabetes medicines on national essential medicines lists (NEMLs) is a first step in achieving adequate access. This selection was studied among LMICs and influences of various factors associated with selection decisions were assessed.

**Methods:**

Countries were studied if they employed NEMLs for reimbursement or procurement purposes. Presence and number of essential diabetes medicines from different classes, both insulins and oral blood glucose lowering medicines, were surveyed and calculated. Data were also analyzed by country income level, geographic region, year of last update of the NEML and purpose of NEML employment. The effect of prevalence and burden of disease on the number of essential diabetes medicines was also studied. Non parametric tests and univariate linear regression analysis were used.

**Results:**

Nearly all countries (n = 32) had chosen fast (97%) and intermediate acting insulin (93%), glibenclamide and metformin (100% both) as essential medicines. The median number of essential diabetes medicines was 6, equally divided between insulins and oral medicines. 20% of the countries had selected insulin analogues as essential medicines. Among all the studied factors, an increase in burden of diabetes and wealth of countries were associated with selection of higher numbers of essential diabetes medicines (p = 0.02 in both cases).

**Conclusions:**

Nearly all the studied LMICs had included the minimum required medicines for diabetes management in their NEMLs. Selection can still be improved (e.g. exclusion of insulin analogues and replacement of glibenclamide by gliclazide). Nevertheless, the known suboptimal and inconsistent availability of essential diabetes medicines in LMICs cannot be explained by inadequate selection of essential medicines. Countries should therefore be encouraged to give precedence to implementation of NEMLs to make essential diabetes medicines more accessible.

## Background

Diabetes is a growing concern in both developed and developing countries. According to the World Health Organization (WHO), 350 million people worldwide have diabetes of whom over 80% live in low and middle income countries (LMICs). The WHO projects that diabetes deaths will double between 2005 and 2030 [1].

In developing countries diabetes care and access to diabetes medicines is a challenge [2]. In some LMICs the life expectancy for a child diagnosed with type 1 diabetes is less than 1 year. (3) Concerns have also been expressed regarding access to type 2 diabetes care especially because its incidence is rapidly growing in LMICs [3]. Complications of diabetes have been reported to cause a massive burden to the African societies, with over 20% retinopathy in newly diagnosed type 2 diabetes patients, around 20% uncontrolled nephropathy in type 1 diabetes patients and many deaths due to undiagnosed diabetes [4]. Studies from India, with the second highest number of diabetes patients globally, also demonstrated suboptimal diabetes care resulting in poor health care outcomes [5], [6].

Sustainable access to medicines for non-communicable disease (NCDs) including diabetes has been pledged by the authorities in the UN resolution of Political declaration of the High-level Meeting of the General Assembly on the Prevention and Control of Non-communicable Diseases [7]. A number of goals have been set and plans have been proposed for countries in a global action plan drafted by the WHO in order to improve the situation till 2020 [8]. Examples of these goals are “Halt the rise in diabetes and obesity”, “10% relative reduction in prevalence of insufficient physical activity” and more relevantly “80% availability of the affordable basic technologies and essential medicines, including generics, required to treat major non-communicable diseases in both public and private facilities”.

Essential medicines are medicines which satisfy the priority health care needs of societies and are considered as a basis for public procurement or reimbursement decisions [9], [10]. As a first step in achieving access to equitable care, it is important to have a rational selection of essential medicines on national essential medicines lists (NEMLs). This can fulfil the prime priorities in health care while dealing with restrictions in health care budgets.

We examined if suboptimal diabetes care outcomes in LMICs stem from inadequate selection of diabetes medicines in NEMLs and if there are differences between LMICs in this respect. In the current study we therefore surveyed the selection of essential diabetes medicines on NEMLs in LMICs. First, selection of essential medicines in different classes of diabetes medicines was studied across these countries. Secondly, the influence of several associating factors including socioeconomic determinants, the burden of disease, prevalence of disease, the purpose of NEML employment and the year of last update of the NEML on selection of diabetes medication on NEMLs was explored.

## Methods

### Data collection and classification

LMICs were eligible for this study if they had responded positively to the questions on the purpose of employment of their NEML (for public procurement, public reimbursement or private insurance purposes) in the Pharmaceutical Country Profile survey conducted by the WHO in 2011 [11], [12]. The latest available update of the NEMLs in these countries was obtained from the “WHO database of essential medicine lists and formularies” [13]. The 32 countries which were included in this study are listed in Table S1 in File S1.

Medicines were included in the study if they were categorized as “medicines used in diabetes” in the NEMLs (or equivalent terms in different NEMLs or languages). The medicines were classified according to the Anatomical Therapeutic Chemical (ATC) classification system [14]. Medicines used in diabetes belong to the ATC category A10, with two main subcategories; A10A “*insulins and analogues*” and *A10B* “*blood glucose lowering drugs, excl. insulins*”. Hereafter the latter group is called “oral blood glucose lowering medicines”. An overview of all medicines used in diabetes and their respective ATC classifications can be found in Table S2 in File S1. Insulins were further categorized as insulin analogues (lispro, aspart, glulisine, glargine, detemir and degludec) or conventional insulins. Insulin analogues are either fast acting or (ultra) long acting insulin types which are more expensive than conventional insulins [15]. Conventional insulins were subsequently classified into recombinant human insulins or natural (animal extracted) insulins. Insulin was only considered recombinant human insulin if this source of insulin was explicitly stated in the NEMLs.

The burden of diabetes in terms of morbidity was obtained for each country from the Institute for Health Metrics and Evaluation (IHME) database [16]. Disease burden was measured as “years lived with disability (YLD)” for the year 2010. The disease burden for diabetes was expressed as the fraction of YLDs attributable to diabetes among total YLDs due to all diseases (for 175 different diseases). Prevalence of diabetes for each country was obtained from the International Diabetes Federation (IDF) estimates for 2010 [17].

Data on geographic regions, income levels and gross domestic product (GDP) per capita were obtained from the WHO and the World Bank, respectively [18], [19]. NEMLs released in 2009 and afterwards were compared with NEMLs dated prior to 2009 to study the influence of revision of the list. In this year a revision of the WHO model list of essential medicines was published. Countries are usually inclined to update their NEMLs subsequent to these revisions.

### Data analysis

Presence and total number of essential medicines for diabetes in the NEMLs of the studied countries and number of ATC categories and subcategories were calculated and analyzed according to the aforementioned classifications. Countries were stratified by the categorical variables, i.e. different WHO geographic regions, the latest World Bank income group classifications and year of NEML update (<2009 vs. >2009). Comparisons regarding the presence and quantity of medicines in different clusters of countries were done with non-parametric tests (Mann–Whitney *U*, Kruskal Wallis and Chi-square tests). Univariate linear regression analysis was used to examine associations between continuous variables (burden of diabetes, disease prevalence and GDP of the countries) versus the total number of essential medicines for diabetes on the NEMLs of the studied countries. Significance level was 0.05 for all the analysis performed. All statistical analyses were conducted using SPSS software, version 19.

## Results

### Selection of essential medicines

**Figure 1:**
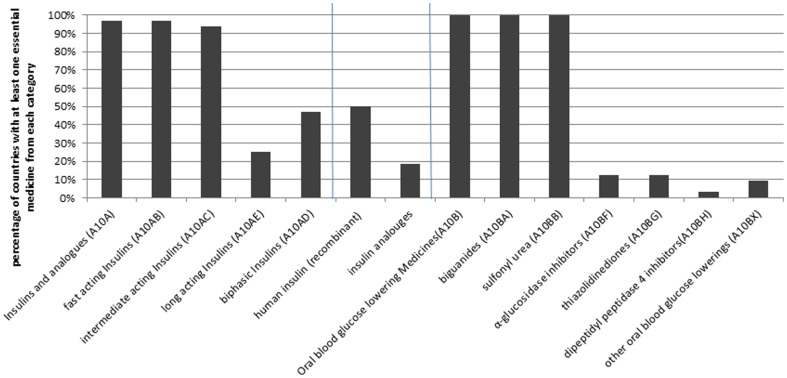
Inclusion of diabetes medications in the NEMLs of low and middle income countries (n = 32).

The overall median number of essential medicines selected for diabetes was 6 medicines, equally divided between “Insulins and analogues” and “Oral blood glucose lowering medicines” (Table 1). Overall, nearly all the studied countries (97%) had at least one product from the “Insulins and analogues” category selected as essential medicine. Almost all the studied countries had selected a fast acting and an intermediate acting insulin for their NEML (97% and 94%, respectively). However, combined insulins (fast and intermediate premix) and long acting insulins were less frequently included in the NEMLs (47% and 25%, respectively). All the studied countries had also incorporated a biguanide (metformin) and at least one sulfonylurea derivative (glibenclamide was included in all the countries) in their NEML. Only in a few countries other categories of oral blood glucose lowering medicines were selected as essential medicines (Figure 1).

**Table 1:** Number of selected essential medicines for diabetes on NEMLs in different geographical regions and across different income levels.

		Number of countries studied/total number of LMICs in the region	Median number of essential medicines for diabetes (range)	Median number of “insulins and analogues” (range)	Median number of “Oral blood glucose lowering” medicines (range)
Global		32/139	6 (4–17)	3 (0–9)	3 (2–8)
WHO region*	Africa	7/44	6 (4–7)	3 (1–4)	3 (2–3)
	Americas	9/26	6 (4–13)	3 (2–5)	3 (2–7)
	Eastern Mediterranean	5/18	5 (4–17)	2 (0–9)	2 (2–8)
	Europe	1/21	5	2	3
	South-East Asia	4/11	5 (5–12)	3 (2–5)	2.5 (2–7)
	Western Pacific	6/19	6.5 (6–8)	3 (3–4)	3 (3–4)
Income levels*	Low income	5	6 (4–6)	3 (1–3)	3 (2–3)
	Lower Middle income	13	5 (4–8)	2 (0–3)	2 (2–5)
	Upper Middle income	14	6.5 (4–17)	3.5 (2–9)	3 (2–8)

*: p values were 0.386, 0.663, 0.865 across different WHO regions and 0.008, 0.008, 0.018 across different income levels for the number of essential medicines for diabetes, number of “Insulin and analogues” and number of “Oral blood glucose lowering” medicines, respectively.

Half of the countries included at least one recombinant human insulin in their NEML while one third (none of the low income countries) had exclusively chosen recombinant human insulin(s). Half of the countries which incorporated solely recombinant human insulin(s) were from the region of the Americas. However, it is important to mention that 14 countries (44%) had not specified the source of insulin in their NEML. Six countries (19%) had selected insulin analogues as essential medicines, all of which were amongst the upper middle income countries and predominantly from the region of the Americas (4 out of 6 countries).

Other classes of oral blood glucose lowering medicines (except biguanides and sulfonylurea derivatives) were primarily selected by upper middle income countries, four of which selected medicines from more than one subcategory of those medicines (Table S3 in File S1). While over half of the countries selected at least 2 medicines as essential medicine from the subcategory of sulfonylurea derivatives, 60% of the lower middle income countries only selected one medicine, namely glibenclamide (p-value  = 0.033).

### Factors associated with selection of essential medicines

When different income levels of the countries were taken into account, the median number of essential medicines for diabetes (and both subcategories) was significantly different across income levels with lower middle income countries having the lowest median number of the essential medicines. The p-values were 0.008, 0.008 and 0.018 across different income levels for number of essential medicines for diabetes, number of “Insulins and analogues” and number of “Oral blood glucose lowering medicines”, respectively (Table 1). No significant differences were found in the number of diabetes essential medicines when results were stratified by geographic region (see Table 1), purpose of NEML employment (procurement or reimbursement) or the NEML's publication date.

**Figure 2:**
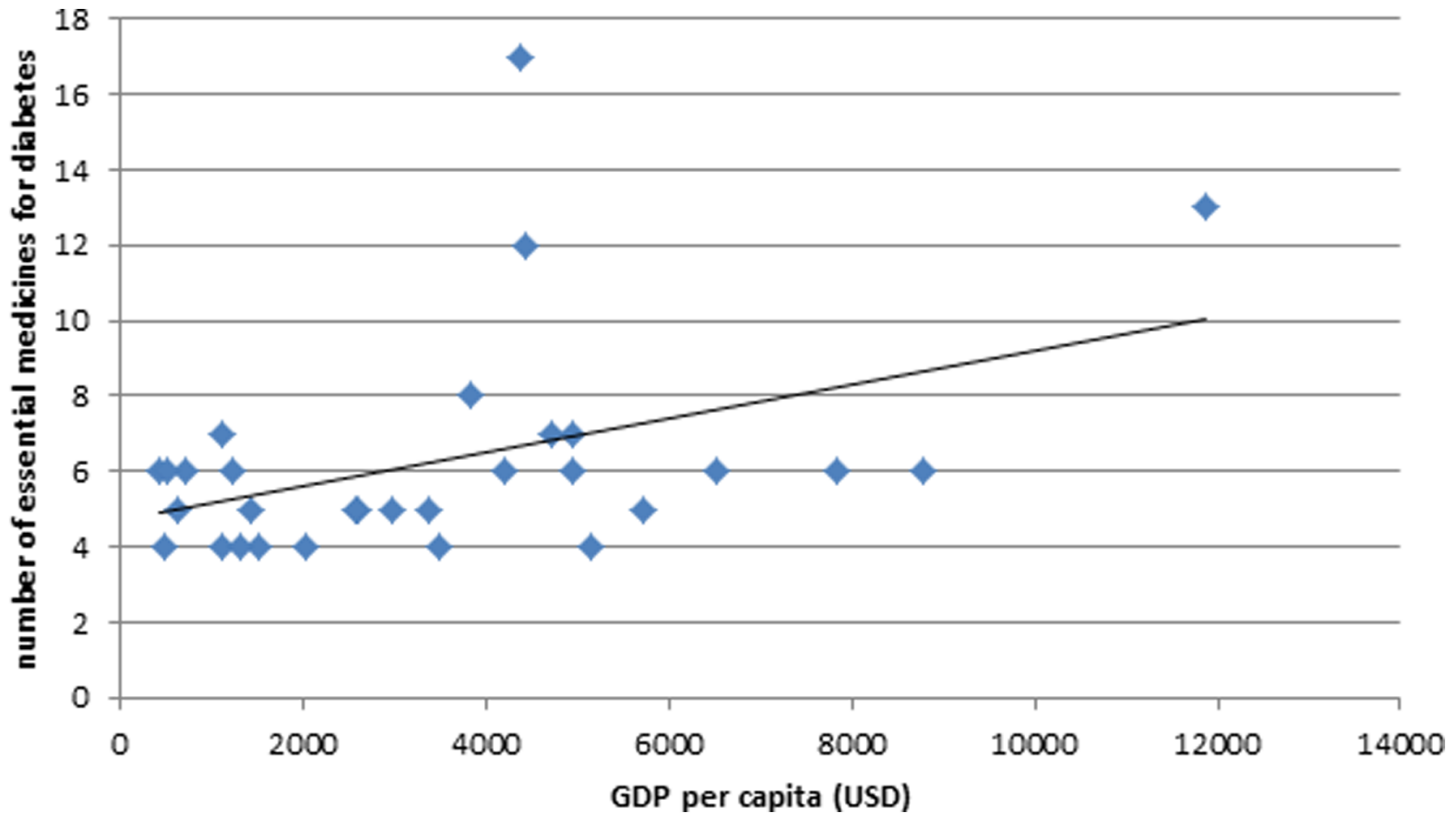
Association between gross domestic product (GDP) per capita and the total number of essential diabetes medicines selected on their NEML by each country in the study.

**Figure 3:**
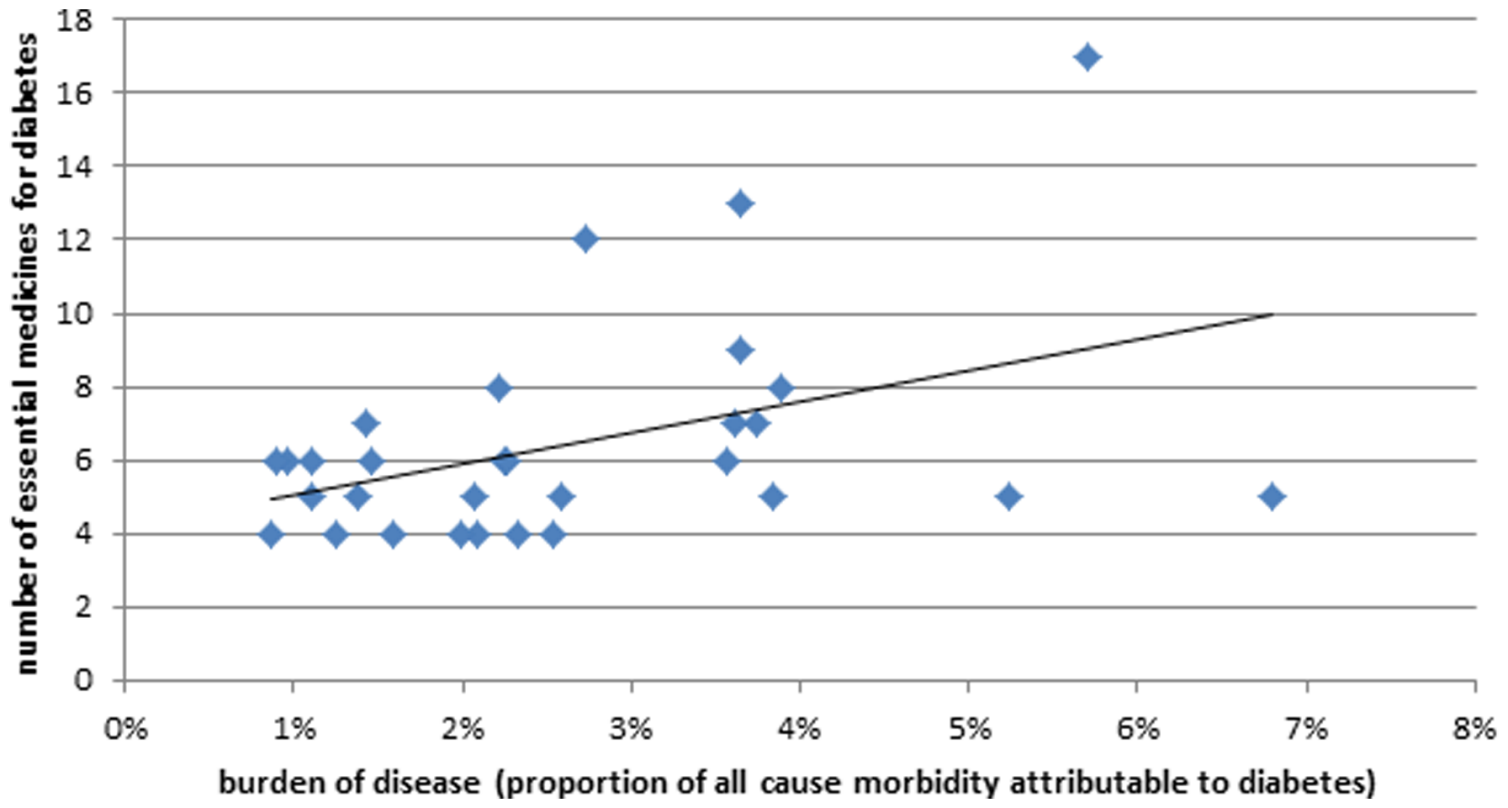
Association between relative burden of diabetes in countries and the total number of essential diabetes medicines selected on their NEML by each country in the study.

An association was observed between GDP per capita of a country and the total number of essential diabetes medicines (β = 0.0005, p-value  = 0.019, Figure 2). The data imply that an increase in GDP per capita by US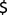 10,000 will on average result in an addition of 5 essential medicines to an NEML for diabetes. The relative burden of diabetes was also associated with the total number of essential medicines (β = 0.8403, p-value  = 0.020, Figure 3), while prevalence of diabetes was not associated (β = 0.1029, p-value  = 0.576). This association with relative burden of diabetes indicates that 1% increase in relative burden of diabetes would on average correspond to inclusion of roughly 1 additional essential medicine for diabetes to a NEML. Similar associations were observed for “oral blood glucose lowering medicines” but not for “Insulins and analogues”.

## Discussion

Diabetes is the fastest growing disease among all NCDs [20]. Long term consequences and secondary complications of diabetes cause a massive burden to societies mainly due to morbidities and productivity loss [21]. Owing to the growing occurrence of diabetes, diabetes care has attracted substantial attention from health care decision makers all around the globe [22]. Therefore, it is important to evaluate access to medicines as a part of diabetes care, especially in LMICs. To our knowledge this is a first (global) attempt to study the factors associated with the selection and the choices made regarding essential medicines for diabetes in LMICs.

In the current study, both “Insulins and analogues” (at least fast and intermediate acting) and “Oral blood glucose lowering medicines” (most frequently metformin and glibenclamide) were selected as essential medicines by almost all surveyed LMICs. A median of three essential medicines from each group were included in the NEMLs for diabetes care. Other classes of oral anti-diabetic medicines as well as insulin analogues were (almost) exclusively selected by upper middle income countries. Associations were identified between GDP per capita of a country as well as relative burden of diabetes and the total number of essential medicines for diabetes on NEMLs. The purpose of employment and year of last update of the NEML as well as prevalence of diabetes had no significant influence on the number of essential medicines selected for diabetes.

In the WHO model list of essential medicines, fast acting and intermediate acting insulin as well as a biguanide (metformin; as an insulin sensitizer) and a sulfonylurea derivative (gliclazide; as an insulin secretagogue) are selected for diabetes management [23]. In the current study, nearly all the studied countries had at least included those medicines in their NEMLs. Selection in these countries therefore generally followed the international guidelines; insulin has a central role in type 1 and in later stages of type 2 diabetes [24], [25]. Besides, there is a broad consensus on metformin as first line treatment and sulfonylurea derivatives as a complementary option in type 2 diabetes treatment [26]–[29]. From a clinical view point, it is reassuring that the first and second line guideline recommended medicines [25], [30], [31] for which data on clinically relevant end points (including cardiovascular disease) exist were covered by nearly all the countries [32]–[34]. Other classes of diabetes medicines were only selected by a few (mainly upper middle income) countries. Lack of evidence on cardiovascular outcomes and higher costs might have impacted the selection decisions [35], [36].

Insulin analogues are not included in the WHO model list and have been critically appraised [37]. In nearly half of the studied upper middle income countries insulin analogues were included in the NEMLs, which were predominantly intended for public reimbursement purposes. According to the WHO report, no clear advantage (with lack of clinically important benefits) over recombinant human insulin has been established [37]. Particularly in the absence of glucose self-monitoring, the benefits of insulin analogues is believed to be minimal [38]. Besides, concerns regarding cost effectiveness have been expressed even in developed countries [15]. The WHO and IDF have argued that spending a substantial share of medical budgets on insulin analogues as seen in some LMICs may indicate an inefficient allocation of constrained health care resources [39]. Reimbursement of insulin analogues and subsequent non guideline-compliant practices incurred roughly £600 million to the National health Services (NHS) in the UK over a decade [15]. This figure - despite belonging to a high income country - can provide an estimation for the dimension of the issue.

According to a Cochrane review, recombinant human insulin is the insulin of choice for new cases of diabetes mellitus [40]. Half of the studied LMICs (particularly low income countries) as well as the WHO for its model list did not specifically select recombinant human insulin as essential medicine. Economic considerations cannot explain this divergence, since there is no concrete evidence to suggest that natural insulin is less costly compared to recombinant human insulin at the time being [41]–[43]. Furthermore, claims of a more predictable response, less allergic reactions and less sensitivity to high temperature of human insulins do not translate to a more favorable benefit risk ratio. This may justify the choice of those countries deciding not to specify between the two insulin types. Regardless of the source, long acting and combined (biphasic or premixed) insulins are believed to have no significant added therapeutic benefit over short and intermediate acting insulins [44], [45]. Therefore, the fact that many countries (as well as the WHO) have limited their choice to the latter types of insulin is justifiable.

The preference has been given to gliclazide in the latest update of the WHO model list for the choice of sulfonylurea derivative. In the current study glibenclamide was selected in all the NEMLs while glicazide was included in slightly over one-third of the NEMLs. Due to its long half-life, glibenclamide has shown the highest rate of hypoglycemia among the second generation of sulfonylurea derivatives which is of concern particularly for the elderly [46], [47]. LMICs may consider prioritizing gliclazide over glibenclamide in their selection for this reason.

The finding that a higher burden of diabetes in countries is associated with a higher number of selected essential diabetes medicines suggests, at least partly, a rational selection procedure. Since a similar association was not observed for prevalence of diabetes, one may conclude that especially severity of the condition has influenced the decisions to select essential medicines for the NEMLs. Given the low explained variance of the relation between burden of diabetes and selection of diabetes medicines further research is necessary to understand selection procedures in countries.

**Figure 4:**
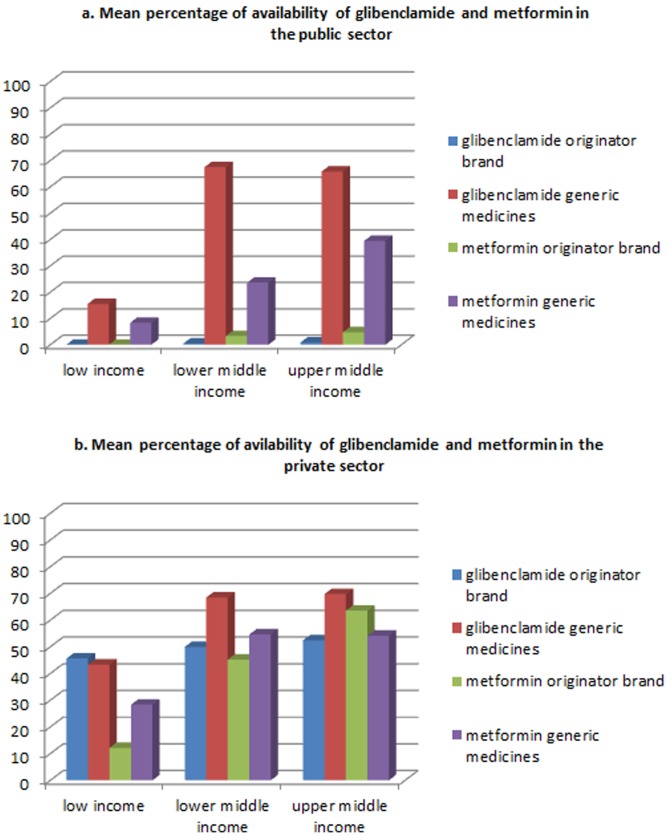
Availability of glibenclamide and metformin across different income levels in the public sector (**figure 4a**) and private sector (**figure 4b**). Data source: Health Action International; Database of survey data, national reports, survey tools and other resources; available at http://www.haiweb.org/medicineprices/.

Few attempts have been made to explore availability of diabetes medicines. Availability of insulin has been surveyed in six LMICs and varied from 20% to 100% across the countries [48]. Mali (low income) and Nicaragua (lower middle income) with 20% and 100% availability, respectively, were also involved in the current study. In a study by Cameron et al. mean availability of oral blood glucose lowering medicines (namely glibenclamide and metformin) was 49.5% and 65.0% in the public and the private sectors of 40 LMICs studied [49]. Part of these countries (17 countries) were also included in the current study, for which the availability was 42.9% and 58.7% for the generic medicines in the public and the private sector, respectively. Further details are provided in Figure 4. As far as affordability is concerned, lack of availability in the public sector and a subsequent shift towards the private sector would impose direct financial burden to the patients. In case of insulin this might incur unbearable expenses to a household which are estimated up to 20 days of wage per month for the lowest-paid government worker [50].

As previously mentioned, rational selection of essential medicines is only the first step in achieving equitable access to medicines. According to the above figures, essential medicines are not adequately available and affordable despite being selected on the NEMLs. This suggests that NEMLs have not been properly implemented yet. According to the WHO framework for access, other elements including sustainable financing, reliable health and supply systems and affordable prices are deemed necessary to enforce essential medicines and ultimately ensure a sustainable access [51]. For instance in case of affordable prices, competition amongst generic medicines has lowered the price of essential oral blood glucose lowering medicines to below 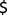
1 a month [52]. However, lack of competition in case of insulin has contributed to unaffordability of such a lifesaving medicine [53]. Further in depth studies from a health system perspective may assist countries in identifying hurdles in the implementation of the essential medicines concept resulting in better access to diabetes care.

This study has some limitations as well. It was conducted at a national level through exploring NEMLs. However, subnational medicines lists and formularies also deserve attention. Medicines might be procured at provincial or even at health care center level differently. In particular insulin products might be provided from various funding resources both nationally and internationally. This requires a case by case situation analysis of each country. Hence, a global survey in particular regarding availability and affordability of insulin is deemed necessary. The Rapid Assessment Protocol for Insulin Access (RAPIA) developed by the International Insulin Foundation (IIF) can be used as a standard protocol to enable further international comparisons [54].

The relatively low number of countries studied could be considered as a general limitation for subgroup analyses where the statistical power was not enough to detect small differences. However, this was due to the limited number of countries that stated the purpose of their NEML employment (either for procurement or reimbursement) in the WHO pharmaceutical country profile project at the time of our study. This was an important inclusion criteria which enabled us to verify if the NEML has implications for access to essential medicines based on self-declaration of the countries. Furthermore, we have not looked at the procedures in place for selection and establishing the NEMLs. A qualitative study of this kind could be complimentary to the current study in order to provide a thorough insight from a health system perspective.

Diabetes care is not limited to medication. Several steps can precede or coincide with medicines, including life style management and modification (e.g. regular physical activities, maintain healthy body weight and healthy diet), diabetes screening in vulnerable groups and timely diagnosis. Self-monitoring as well as clinical monitoring is of utmost importance in order to avoid or delay further complications [1]. Besides, access to treatment encompasses more aspects than just access to medicines. This includes paying due attention to - among others - cultural barriers, availability of trained medical staff, and adequate access to insulin syringes and blood glucose test strips [55]. Studies are required to assess the entire diabetes care plans of different countries and measure their clinical outcomes. This would result in a comprehensive evaluation of diabetes care within which pharmaceutical care can be better positioned. National and regional studies have been conducted in this respect, but global studies are needed [56]–[58].

In conclusion, Nearly all the studied LMICs (regardless of region and socio-economic status among others) have included the minimum essential requirements for the pharmacological treatment of type 1 and type 2 diabetes in their NEMLs. Wealth of countries and burden of diabetes are recognized to partially influence the number of medicines designated essential for diabetes. As far as selection is concerned, constrained health care resources might be better utilized by reconsideration of choices made (e.g. exclusion of insulin analogues, replacement of glibenclamide by gliclazide).Studies evaluating access to diabetes medicines do not support adequate availability of essential medicines for diabetes. Therefore precedence should be given to the implementation of NEMLs in order to increase sustainable access to essential diabetes medicines in LMICs.

## Supporting Information

File S1 **Table S1**, Countries and surveys included in the study. **Table S2**, ATC classification for diabetes medicines. **Table S3**, Countries which have selected other classes of oral blood glucose lowering agents besides biguanides and sulfonylurea derivatives on their NEML.
(DOCX)
