# Supplementary material for: Selection of Essential Medicines for Diabetes in Low and Middle Income Countries: A Survey of 32 National Essential Medicines Lists
Source: PLoS One. 2014 Sep 26;9(9):e106072. doi: 10.1371/journal.pone.0106072 (PMC4178014; doi:10.1371/journal.pone.0106072)
Supplement: File S1 — Table S1, Countries and surveys included in the study. Table S2, ATC classification for diabetes medicines. Table S3, Countries which have selected other classes of oral blood glucose lowering agents besides biguanides and sulfonylurea derivatives on their NEML. (DOCX) [file pone.0106072.s001.docx]

**Table S1. Countries and surveys included in the study**

| **Country** | **WHO Region** | **Income level** | **NEML publication year** |
| --- | --- | --- | --- |
| Armenia | Europe | lower middle income | 2010 |
| Argentina | America | upper middle income | 2005 |
| Bolivia | America | lower middle income | 2011 |
| Cameroon | Africa | lower middle income | 2009 |
| Central African Republic | Africa | low income | 2009 |
| Chad | Africa | low income | 2007 |
| China | Western Pacific | upper middle income | 2009 |
| Ecuador | America | upper middle income | 2009 |
| Egypt | Eastern Mediterranean | lower middle income | 2006 |
| El Salvador | America | lower middle income | 2009 |
| Eritrea | Africa | low income | 2010 |
| India | South-East Asia | lower middle income | 2011 |
| Jamaica | America | upper middle income | 2008 |
| Jordan | Eastern Mediterranean | upper middle income | 2011 |
| Malaysia | Western Pacific | upper middle income | 2008 |
| Maldives | South-East Asia | upper middle income | 2011 |
| Mali | Africa | low income | 2008 |
| Morocco | Eastern Mediterranean | lower middle income | 2008 |
| Namibia | Africa | upper middle income | 2008 |
| Nicaragua | America | lower middle income | 2011 |
| Pakistan | Eastern Mediterranean | lower middle income | 2007 |
| Palau | Western Pacific | upper middle income | 2006 |
| Peru | America | upper middle income | 2010 |
| Solomon Islands | Western Pacific | lower middle income | 2010 |
| Sri Lanka | South-East Asia | lower middle income | 2009 |
| Sudan | Eastern Mediterranean | lower middle income | 2007 |
| Suriname | America | upper middle income | 2004 |
| Thailand | South-East Asia | upper middle income | 2008 |
| Tonga | Western Pacific | lower middle income | 2007 |
| Tuvalu | Western Pacific | upper middle income | 2008 |
| Uganda | Africa | low income | 2007 |
| Uruguay | America | upper middle income | 2011 |

**Table S2. ATC classification for diabetes medicines**

| **A10A** | **Insulins and analogues** |  |  |
| --- | --- | --- | --- |
|  | **A10AB** | **Insulins and analogues for injection, fast-acting** |  |
|  |  | **A10AB01** | **Insulin (human)** |
|  |  | **A10AB02** | **Insulin (beef)** |
|  |  | **A10AB03** | **Insulin (pork)** |
|  |  | **A10AB04** | **Insulin lispro** |
|  |  | **A10AB05** | **Insulin aspart** |
|  |  | **A10AB06** | **Insulin glulisine** |
|  |  | **A10AB30** | **Combinations** |
|  | **A10AC** | **Insulins and analogues for injection, intermediate-acting** |  |
|  |  | **A10AC01** | **Insulin (human)** |
|  |  | **A10AC02** | **Insulin (beef)** |
|  |  | **A10AC03** | **Insulin (pork)** |
|  |  | **A10AC04** | **Insulin lispro** |
|  |  | **A10AC30** | **Combinations** |
|  | **A10AD** | **Insulins and analogues for injection, intermediate-acting combined with fast-acting** |  |
|  |  | **A10AD01** | **Insulin (human)** |
|  |  | **A10AD02** | **Insulin (beef)** |
|  |  | **A10AD03** | **Insulin (pork)** |
|  |  | **A10AD04** | **Insulin lispro** |
|  |  | **A10AD05** | **Insulin aspart** |
|  |  | **A10AD30** | **Combinations** |
|  | **A10AE** | **Insulins and analogues for injection, long-acting** |  |
|  |  | **A10AE01 Insulin (human)** | **Insulin (human)** |
|  |  | **A10AE02 Insulin (beef)** | **Insulin (beef)** |
|  |  | **A10AE03 Insulin (pork)** | **Insulin (pork)** |
|  |  | **A10AE04** | **Insulin glargine** |
|  |  | **A10AE05** | **Insulin detemir** |
|  |  | **A10AE30** | **Combinations** |
|  | **A10AF** | **Insulins and analogues for inhalation** |  |
|  |  | **A10AF01** | **Insulin (human)** |
| **A10B** | **Blood glucose lowering drugs, excluding insulins** |  |  |
|  | **A10BA** | **Biguanides** |  |
|  |  | **A10BA01** | **Phenformin** |
|  |  | **A10BA02** | **Metformin** |
|  |  | **A10BA03** | **Buformin** |
|  | **A10BB** | **Sulfonamides, urea derivatives** |  |
|  |  | **A10BB01** | **Glibenclamide** |
|  |  | **A10BB02** | **Chlorpropamide** |
|  |  | **A10BB03** | **Tolbutamide** |
|  |  | **A10BB04** | **Glibornuride** |
|  |  | **A10BB05** | **Tolazamide** |
|  |  | **A10BB06** | **Carbutamide** |
|  |  | **A10BB07** | **Glipizide** |
|  |  | **A10BB08** | **Gliquidone** |
|  |  | **A10BB09** | **Gliclazide** |
|  |  | **A10BB10** | **Metahexamide** |
|  |  | **A10BB11** | **Glisoxepide** |
|  |  | **A10BB12** | **Glimepiride** |
|  |  | **A10BB31** | **Acetohexamide** |
|  | **A10BC** | **Sulfonamides (heterocyclic)** |  |
|  |  | **A10BC01** | **Glymidine** |
|  | **A10BD** | **Combinations of oral blood glucose lowering drugs** |  |
|  |  | **A10BD01** | **Phenformin and sulfonamides** |
|  |  | **A10BD02** | **Metformin and sulfonamides** |
|  |  | **A10BD03** | **Metformin and rosiglitazone** |
|  |  | **A10BD04** | **Glimepiride and rosiglitazone** |
|  |  | **A10BD05** | **Metformin and pioglitazone** |
|  |  | **A10BD06** | **Glimepiride and pioglitazone** |
|  |  | **A10BD07** | **Metformin and sitagliptin** |
|  |  | **A10BD08** | **Metformin and vildagliptin** |
|  |  | **A10BD09** | **Pioglitazone and alogliptin** |
|  |  | **A10BD10** | **Metformin and saxagliptin** |
|  |  | **A10BD11** | **Metformin and linagliptin** |
|  |  | **A10BD12** | **Pioglitazone and sitagliptin** |
|  |  | **A10BD13** | **Metformin and alogliptin** |
|  | **A10BF** | **Alpha glucosidase inhibitors** |  |
|  |  | **A10BF01** | **Acarbose** |
|  |  | **A10BF02** | **Miglitol** |
|  |  | **A10BF03** | **Voglibose** |
|  | **A10BG** | **Thiazolidinediones** |  |
|  |  | **A10BG01** | **Troglitazone** |
|  |  | **A10BG02** | **Rosiglitazone** |
|  |  | **A10BG03** | **Pioglitazone** |
|  | **A10BH** | **Dipeptidyl peptidase 4 (DPP-4) inhibitors** |  |
|  |  | **A10BH01** | **Sitagliptin** |
|  |  | **A10BH02** | **Vildagliptin** |
|  |  | **A10BH03** | **Saxagliptin** |
|  |  | **A10BH04** | **Alogliptin** |
|  |  | **A10BH05** | **Linagliptin** |
|  |  | **A10BH51** | **Sitagliptin and simvastatin** |
|  | **A10BX** | **Other blood glucose lowering drugs, excluding insulins** |  |
|  |  | **A10BX01** | **Guar gum** |
|  |  | **A10BX02** | **Repaglinide** |
|  |  | **A10BX03** | **Nateglinide** |
|  |  | **A10BX04** | **Exenatide** |
|  |  | **A10BX05** | **Pramlintide** |
|  |  | **A10BX06** | **Benfluorex** |
|  |  | **A10BX07** | **Liraglutide** |
|  |  | **A10BX08** | **Mitiglinide** |
|  |  | **A10BX09** | **Dapagliflozin** |
| **A10X** | **Other drugs used in diabetes** |  |  |
|  | **A10XA** | **Aldose reductase inhibitors** |  |
|  |  | **A10XA01** | **Tolrestat** |

**Table S3. Countries which have selected other classes of oral blood glucose lowering agents besides biguanides and sulfonylurea derivatives on their NEML**

| **Country** | **Region** | **Income level** | **NEML year** | **Other classes of diabetes medicines designated as essential** |
| --- | --- | --- | --- | --- |
| Argentina | America | upper middle income | 2005 | Alpha glucosidase inhibitors (e.g. acarbose) |
| Jamaica | America | upper middle income | 2008 | Alpha glucosidase inhibitors (e.g. acarbose)  Thiazolidinediones (e.g. pioglitazone) |
| Jordan | Eastern Mediterranean | upper middle income | 2011 | Thiazolidinediones (e.g. pioglitazone)  Dipeptidyl peptidase 4 (DPP-4) inhibitors  Other blood glucose lowering drugs(e.g. Repaglinide) |
| Morocco | Eastern Mediterranean | lower middle income | 2008 | Alpha glucosidase inhibitors (e.g. acarbose) |
| Thailand | South-East Asia | upper middle income | 2008 | Alpha glucosidase inhibitors (e.g. acarbose)  Thiazolidinediones (e.g. pioglitazone)  Other blood glucose lowering drugs(e.g. Repaglinide) |
| Uruguay | America | upper middle income | 2011 | Thiazolidinediones (e.g. pioglitazone)  Other blood glucose lowering drugs(e.g. Repaglinide) |
